# Persistent Stapedial Artery, Oval Window Atresia and Congenital Stapes Agenesis—Case Report

**DOI:** 10.3390/medicina59030461

**Published:** 2023-02-25

**Authors:** Dan Cristian Gheorghe, Veronica Epure, Doru Oprea, Adina Zamfir-Chiru-Anton

**Affiliations:** 1 ENT Department, University of Medicine and Pharmacy Carol Davila Bucharest, 050474 Bucharest, Romania; 2 ENT Department, MS Curie Hospital, 077120 Bucharest, Romania; 3 ENT Department, Grigore Alexandrescu Emergency Hospital for Children, 011743 Bucharest, Romania

**Keywords:** persistent stapedial artery, cochleostomy, ossiculoplasty

## Abstract

*Background*: The persistent stapedial artery (PSA) is a rare congenital vascular malformation involving the middle ear. It is usually associated with pulsatile tinnitus and/or conductive hearing loss and can account for multiple risks during middle ear surgery. *Case Report*: we present a case of a 9-year-old male child with conductive hearing loss and persistent stapedial artery in his right ear, who was admitted to our ENT Department for hearing loss. During surgery, we discovered PSA along with congenital stapes agenesis and oval window atresia, as well as an abnormal trajectory of the mastoid segment of the facial nerve. After ossicular reconstruction (transcanal total ossicular replacement prosthesis) with cochleostomy, no surgical complications were recorded and hearing improvement was monitored by pre- and postoperative audiometry. *Conclusion*: Stapedial artery is a rare anatomical middle ear abnormality that can prevent proper surgical hearing restoration and can be associated with other simultaneous temporal bone malformations.

## 1. Introduction

The persistent stapedial artery (PSA) is a rare vascular congenital abnormality originating from the second aortic arch, with a prevalence of 0.02–0.48% (5 out of 1045) as a temporal bone study shows [1]. Across embryonic development it connects the external and internal carotid arteries, only to involute during the 10th week of fetal development [2]. During this time, it shapes the stapes suprastructure as it passes along the promontory and then the obturator foramen, on its course from its origin in the external carotid artery to the middle cranial fossa, usually through a bony dehiscence of the facial canal wall just posterior from the cochleariform process; all the four subtypes of PSA described have in common that the PSA runs over the promontory and courses through the obturator foramen of the stapes to enter the facial canal. After exiting the facial canal just before the geniculate ganglion, the PSA travels anteriorly and cranially into the extradural space of the middle cranial fossa. If the stapedial artery persists into postnatal life, it supplies the middle meningeal artery.

PSA varies anatomically and among the known types there are described: the hyoido-stapedial artery, the pharyngo-stapedial artery, the stapedial artery with aberrant carotid artery and the pharyngo-hyo-stapedial artery, with the first type being the most described in literature. It is often an incidental discovery (discovered on CT scan images or intraoperatively during middle ear surgery), as it is mostly asymptomatic. It can also be associated with tinnitus, conductive hearing loss (by limiting the movement of the stapes, thereby mimicking otosclerosis), unilateral or bilateral pulsatile tinnitus or even dizziness or vertigo [3]. Sensorineural hearing loss has also been reported as a consequence of erosion of the otic capsule [4].

Given the anatomical variations of PSA and its trajectory through the middle ear, the surgeon must consider the risk of facial palsy, hearing loss, hemiplegia and account for the possibility of an aberrant internal carotid artery which can emerge in the hypotympanum without bone protection and can be easily injured during middle ear exploration [5]. Still, the utmost clinical significance is the hemorrhage as a consequence of accidental injury of the vessel. Other middle ear anomalies can be discovered in patients with PSA, from facial nerve anomalies [6,7], various blood vessel anomalies [8], to different stapes deformities [9,10,11].

The golden standard for diagnosing PSA is computed tomography (CT) scan which displays the absence of foramen spinosum and the abnormal widening of the anterior fallopian canal. Angiography, magnetic resonance angiography (MRA) or magnetic resonance imaging (MRI) scan are the only imaging tests which could specifically exhibit the artery and they might prove useful only in selected cases if one considers the variable diameter of this vessel (0.4–2 mm) [3].

We report a case of persistent stapedial artery associated with other rare middle ear malformations in a paediatric patient; informed written consent from the child’s parents and approval from the Hospital’s Ethics Committee were obtained (approval no. 45595/21 October 2022).

## 2. Case Presentation

We present the case of a 6-year-old male child who was first examined in our Department for bilateral hearing loss. 

**Figure 1:**
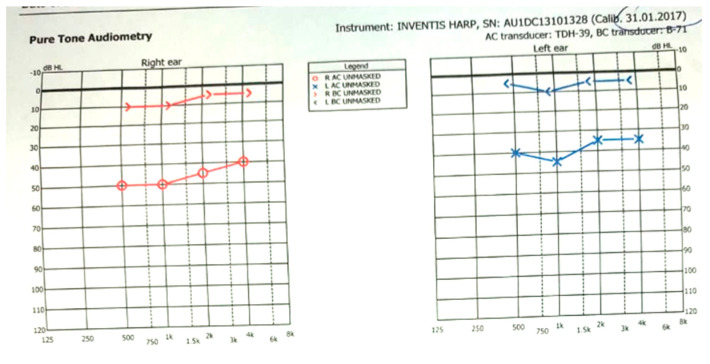
Audiogram from 31 January 2017 (Child age—6 years) showing bilateral conductive hearing loss with air-bone gap, suggesting bilateral middle ear affliction.

From history, the child was born by vaginal delivery, on term, and had no past medical problems. We also made the diagnosis of bilateral mixed hearing loss, with an air-bone gap (ABG) of 35 and 40 dB at 1000 Hz, respectively, and normal bilateral type A tympanogram (Figure 1). As such, the audiological investigations suggested an ossicular chain fixation. 

**Figure 2:**
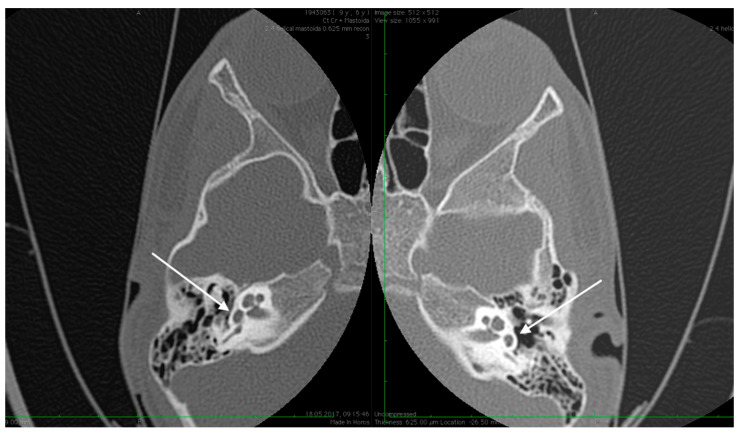
High resolution axial CT scan of left temporal bone showing a missing oval window and stapes suprastructure (arrow) on both sides.

**Figure 3:**
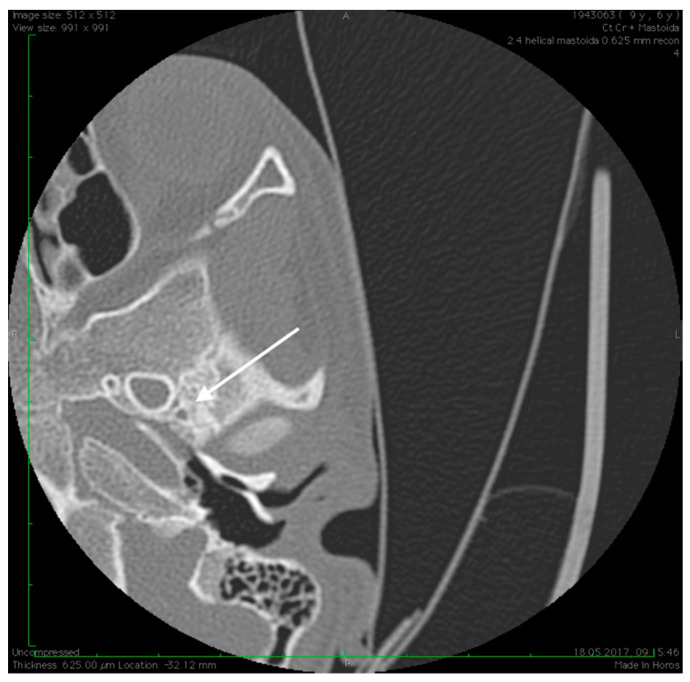
High resolution axial CT scan of left temporal bone exposing the patent foramen spinosum (arrow).

**Figure 4:**
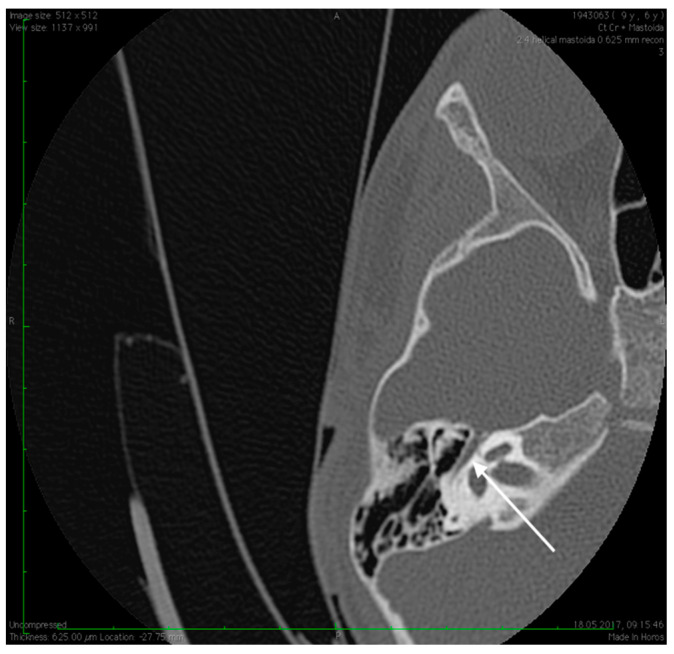
High resolution axial CT scan of right temporal bone exposing the unusual wide facial canal and the missing oval window on the right side (arrow).

**Figure 5:**
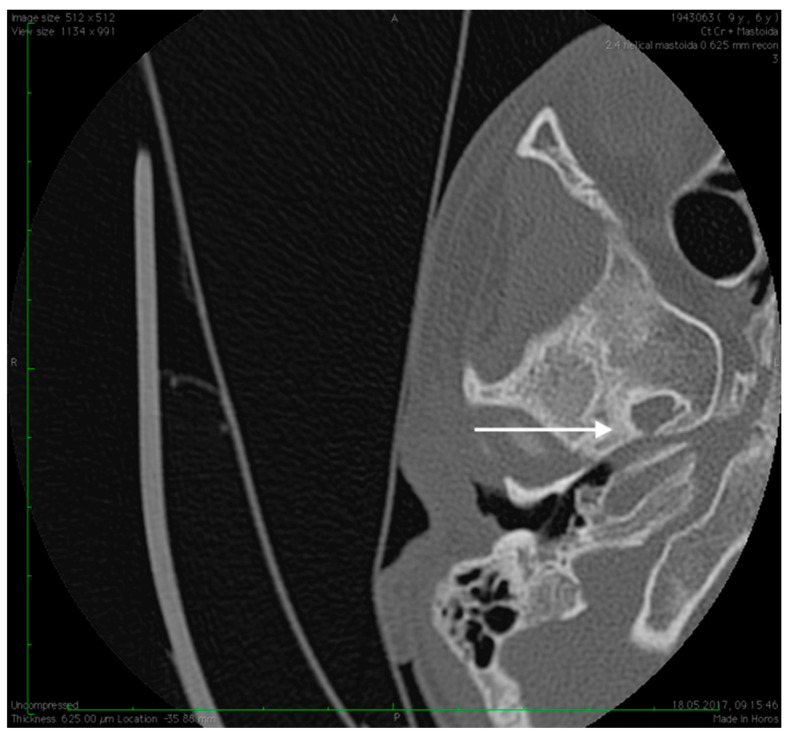
High resolution axial CT scan of right temporal bone exposing the missing foramen spinosum (arrow).

**Figure 6:**
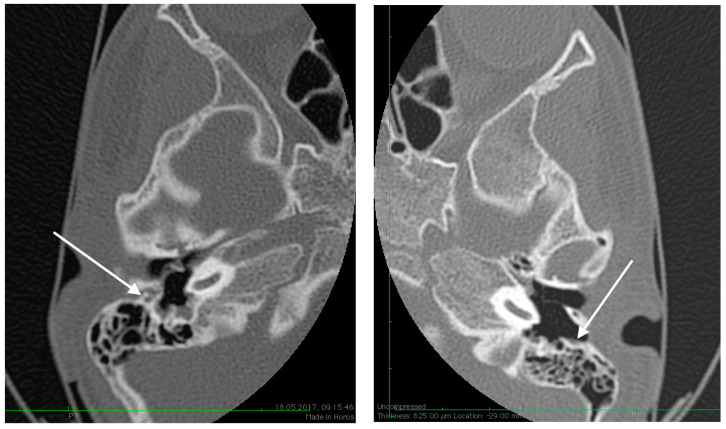
Abnormal position of the facial nerves in their mastoid segment, bilaterally (arrows).

No family history of hearing problems could be elicited. A high resolution CT scan revealed bilateral middle ear anomalies. There were no images of the oval window and stapes suprastructure in his left ear, with abnormal facial nerve canal position and patent foramen spinosum (Figure 2 and Figure 3). The stapes suprastructure and the oval window were also missing to the contralateral (right) ear with a wide facial nerve canal between the geniculate and the round window niche and an abnormal traject on its mastoid segment. No foramen spinosum was observable on the right side (Figure 4, Figure 5 and Figure 6). Furthermore, surgery was carefully planned to investigate the middle ear.

The middle ear exploration of the right ear discovered a bright red pulsatile vessel across the promontory, in a cranial to caudal position, covering the round window and associating the absence of the stapes and the oval window. The diagnostic of persistent stapedial artery (PSA) was made, which resulted in postponing the right ear ossicular chain reconstruction. 

During the following 3 years the child underwent surgical approaches for his left ear hearing loss. Stapes agenesis, but with oval window presence were recorded, with no associated PSA on that side. A stapedotomy and a Teflon wire piston attached to the incus were performed for reconstruction of the ossicular chain. This was followed by two other consecutive ossicular chain release surgeries, due to recurrent bony fixation of the incus and malleus to the middle ear walls, as observed intraoperatively. Hearing in the left ear had later degraded, as the monitoring audiograms showed, with minor improvement of the ABP persisting on long term follow-up (from 51.6 dB preoperatively to 41.6 dB after surgery). 

**Figure 7:**
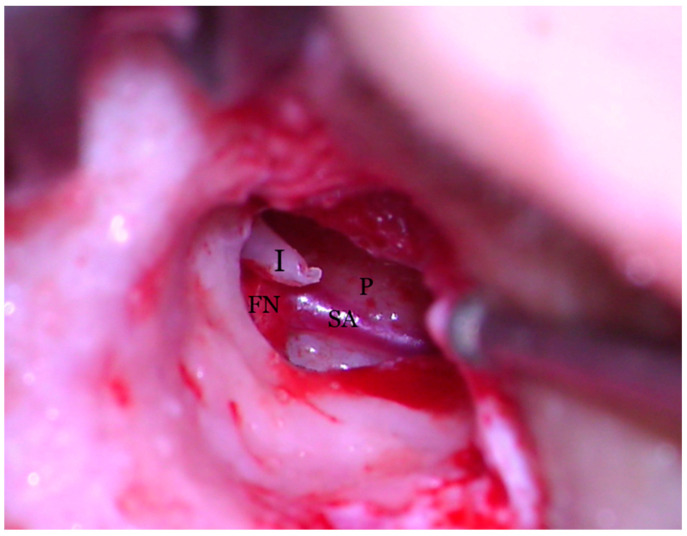
Transcanalar microscopic aspect of right middle ear: the Stapedial Artery (SA) can be seen runnning across the promontory (P) and joining with the facial nerve (FN) right under the incus (I) long process.

**Figure 8:**
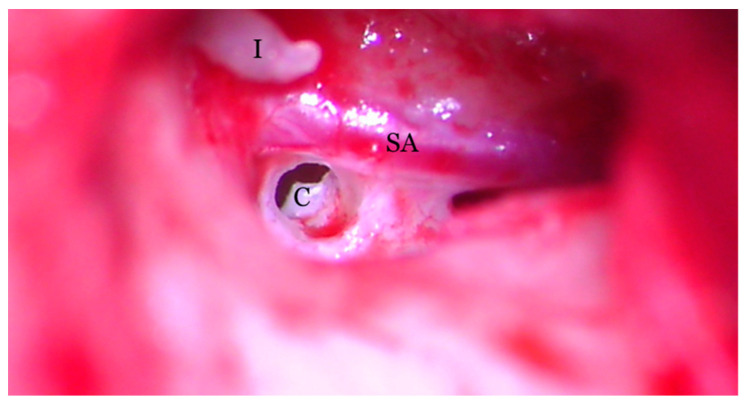
Transcanalar microscopic aspect of the right middle ear: the cochleostomy (C) orifice can be observed in the near vecinity of the stapedial artery (SA) and facial nerve (FN). The long process of the incus (I) can also be observed.

**Figure 9:**
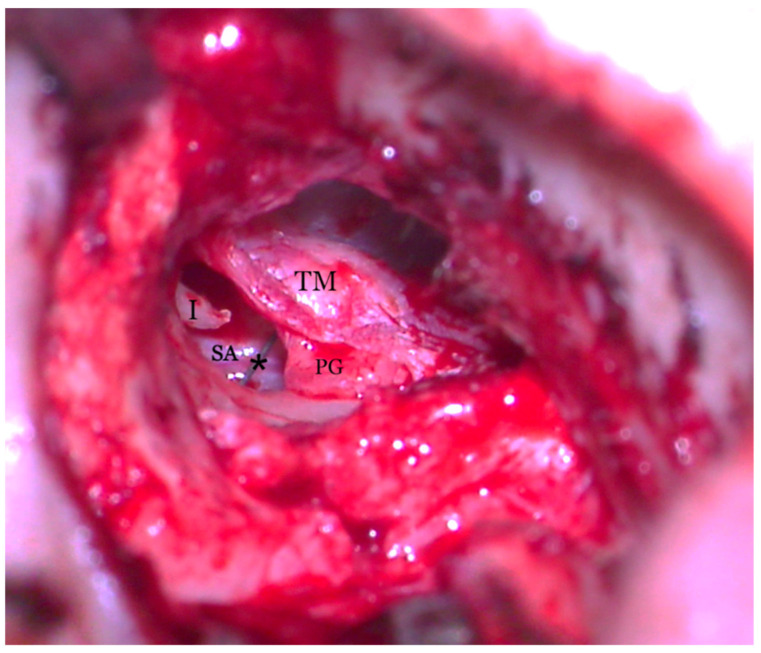
Transcanalar microscopic aspect of middle and external right ear: the handle of the TORP (*) can be observed lying in close contact with the stapedial artery (SA). The perichondrium graft (PG) stands between the plate of the TORP and the tympanomeatal flap (TM). (I) incus long process.

**Figure 10:**
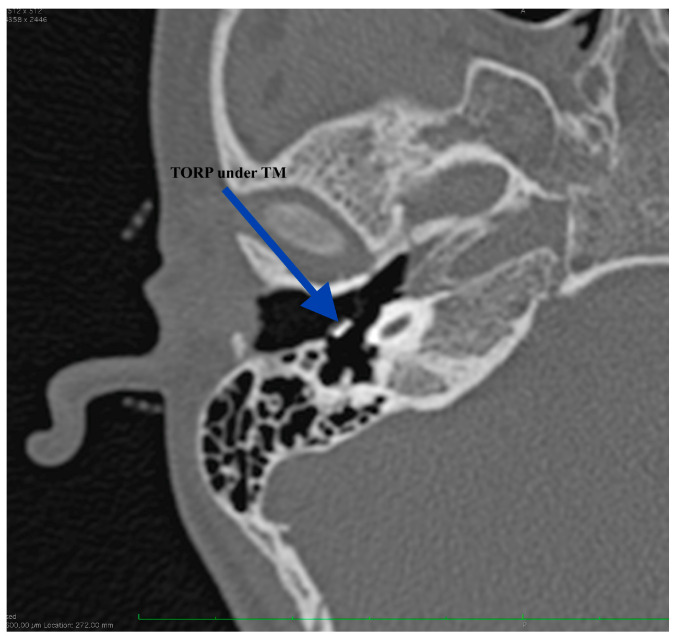
Final aspect of the right ear, with the perichondrium graft (PG) going under the tympanomeatal flap (TM) and sealing the middle ear space.

Hearing loss in patient’s right ear (with PSA) was addressed by performing an ossiculoplasty with a total ossicular replacement prosthesis (TORP). After a thorough preoperative planning based on prior surgical middle ear exploration and CT imaging, a transcanalar approach to the middle ear was used. A standard tympanomeatal flap was raised and the PSA was inspected. Large dimensions, complete coverage of the oval window site and crossing over the round window niche were again noted (Figure 7). The PSA went superiorly across the promontory and entered the facial nerve canal at the level where the normal oval window niche would have been located. Successful cochleostomy was carried out with a diamond burr posteriorly to the PSA and inferiorly to the facial nerve canal. Perichondrium was used to seal the cochlea opening and also served as a support for a total replacement ossicular prosthesis (Figure 8). The TORP was seated in place and covered with a mixed graft of cartilage and perichondrium with the tympanic membrane on top of it (Figure 9 and Figure 10).

**Figure 11:**
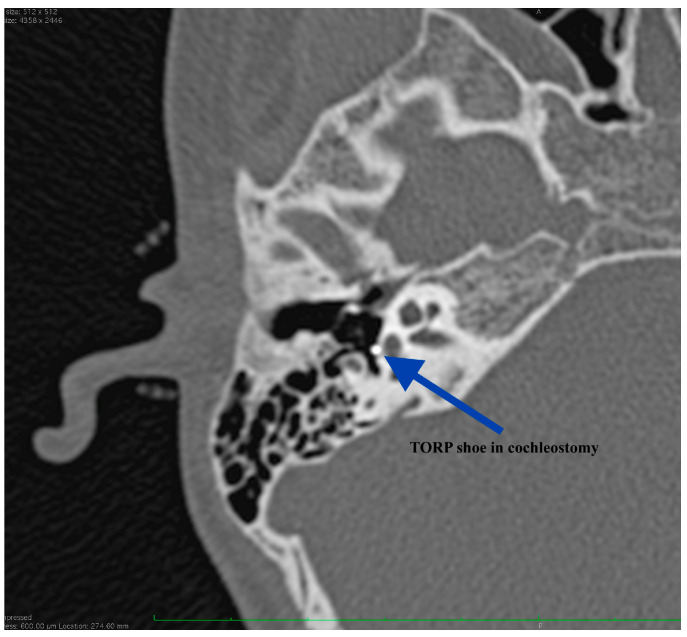
CT scan at 9 months after surgery, showing proper positioning of the prosthesis: TORP shoe in the cochleostomy.

The patient reported immediate postoperative good hearing but failed to present at our Department for performing an audiogram. His hearing was firstly evaluated at 2 months after right ear surgery demonstrating an air-bone gap of 41.6 dB (from 56.6 dB preoperatively). We could not explain at that moment the results but, at 9 months after surgery we were able to have another CT scan performed. The imaging showed good positioning of the ossicular prosthesis (Figure 10 and Figure 11). Without surgical investigation of the middle ear is it impossible to know the precise reason why the hearing restoration is not as good as we expected. We can hypothesize about local healing changes but we cannot be sure.

Future surgical alternative approaches were contemplated in this case for hearing rehabilitation, considering the presented abnormalities, and the patient has been planned for osseointegrated implant surgery (OSIA).

## 3. Discussion 

PSA is a rare condition of the middle ear, with an estimated incidence of 0.02–0.5%, discovered mostly incidentally, thus creating a great impact on surgeons who can be forced to discontinue interventions, such as Govaerts et al. show in their paper [12]. 

It evolves during embryologic stages from the hyoid artery, a branch of the external carotid artery which derives from the pharyngeal artery in the second aortic arch [13]. It runs across the promontory and shapes the stapes by passing between the crura. Above this point, it divides into an upper and a lower division, supplying the dura mater through the middle meningeal artery, the orbit, maxillary and mandibular structure through its other branches [14]. Its passage through the crus of the stapes limits the size of the artery, which can no longer supply the necessary blood to the final structures over a certain body size. This, and the later connection of the internal and external carotid artery through the anastomosis between the maxillomandibular division of the stapedial artery and the distal ventral pharyngeal artery, promote the involution of the stapedial artery in normal humans [15].

Understanding the embryological development of PSA can increase the confidence of handling it, either by cutting or coagulating it with CO_2_ laser or simply avoiding it when possible. In our presented case, the large diameter of the vessel (possibly favored by stapes absence) prevented us from trying to coagulate or manipulate it, thus avoiding a high-risk bleeding that would have made the intervention impossible to be continued or extend it beyond its rehabilitation purposes. The absence of the stapes and the oval window, which would have oriented us towards a stapedotomy, forced us to perform a cochleostomy, while avoiding PSA trajectory.

The multitude of divisions of the stapedial artery pave the way for multiple anatomical variations of the PSA. Apart from this variety of anatomical disposals, literature review demonstrates that PSA is often associated with other middle ear structure deviations, like stapes footplate ankylosis [10,11], malformed stapes suprastructure [13], malpositioned facial nerve [7] or thickened middle ear mucosa that hides the PSA [9]. Particular to our case, we noted the PSA to be accompanied by bilateral congenital stapes agenesis, unilateral oval window atresia and bilateral abnormal facial nerve canal position. Since the first documentation of PSA existence (Hyrtl, 1836) we could only find few literature references about similar conditions, and only in one single reported case (Hoogland and Marres, 1977) PSA was associated with oval window atresia and aberrant stapes suprastructure.

Preoperative CT imaging of middle ear surgery patients is mandatory and careful interpretation of the results can sometimes prevent accidental injuries brought to a PSA. The usual findings are a small, abnormal, canaliculus leaving the carotid canal or the facial nerve, a straight line structure crossing over the promontory, an unusual widening of the fallopian canal or a different canal parallel to it between the geniculate and the oval window niche and absence of the foramen spinosum [16]. Our CT findings coincide with some of the aforementioned signs, as there can be seen no trace of the foramen spinosum in the affected ear (Figure 5), in contrast to the contralateral ear (Figure 3). There is also a widening of the facial nerve canal that can be observed in Figure 4. It came as no surprise that the stapes could not be described in the CT images, as the literature review acknowledges the possible stapes malformations that come along with PSA. 

Even if there are many reports on CT findings and embryologic explanations on persistent stapedial artery, there are few literature data regarding the main surgical approaches to the persistent stapedial artery, as this can be an incidental CT or intraoperative finding complicating middle ear surgery or cochlear implantation, always a great dilemma to the surgeon, who must decide whether to abort or complete the procedure and how to manage the PSA. Successful surgical implantation in the presence of PSA has been described by some authors [17], also reconstruction of the ossicular chain (PORP, TPRP), stapes surgery without transaction of the PSA (stapedectomy with intact PSA—Baron 1963, House 1964, Pahor 1992; stapedotomy with intact PSA—Govaerts 1993, with profuse bleeding or floating footplate as complications, Pirodda 1994; maleostapedotomy with intact PSA—Sugimoto 2014) (reported in 9 ears) [18], stapes surgery combined with transaction of PSA (in 5 operated ears—PSA sectioned by Schweitzer 1984, Yamamoto 1988, Karosi 2008 with minor intraoperative bleeding; PSA transected by laser coagulation or cauterisation by Murphy 1995, Silbergleit 2000, Hitier, Goderie 2013, with intact stapes; clips on PSA by Fisch 1980, Araujo 2002, with intact stapes) have been described [18], sometimes with good hearing outcomes (hearing improvement and subsiding of the pulsatile tinnitus), manageable preoperative bleeding in four patients and no significant postoperative sequelae. Cited complications of surgery in cases of PSA are: profuse or minor intraoperative bleeding (controlled by pressure on the vessel, bipolar coagulation, Tabotamp application), floating footplate with consequent abortion of procedure [18]. 

Being sometimes asymptomatic, the act to manipulate the PSA or avoid it is a decision each surgeon has to take during intervention, based on the patients’ needs and considering the significant risk of permanent hemiplegia, facial nerve paralysis, tabes dorsalis-like symptoms, and hearing loss, even if multiple papers describe successful discontinuations of the artery, as well. Carefully preparing of the surgery cannot rule out the possibility of misinterpreting the presence of the PSA but certain anatomical aspects of the CT imaging specific to the PSA can warn the surgeon of such possible malformation of the middle ear, thus making the approach more cautious. 

Considering our case once again, we could try stapedial artery coagulation but with the risk of neurologic damage or impossibility to achieve useful hemostasis. That would have also incured the presence of a normal oval window, in order to use it for TORP reconstruction. In our case, no such structure existed, leaving just the cochleostomy option as the only approach. We can also mention that the inferior traject of the stapedial artery partially covered the round window niche. Its presence and free movement is also important to consider when functional reconstruction is the target.

## 4. Conclusions

PSA is a rare disease, sometimes difficult to diagnose before surgery, even with imaging. Middle ear surgery in the presence of a PSA is possible without any major complications, but the surgeon alone must decide whether to manipulate the artery from its bed or to coagulate it. High resolution CT imaging is a good tool to assess the possibility of any middle ear associated abnormalities. Dimension of the artery is the main risk factor for final surgical outcome and intraoperative complications. Cochleostomy and ossicular chain reconstruction could represent an effective approach to managing hearing loss in cases with PSA. Different possible types of ossicular chain replacement prothesis could eventually yield better results, in terms of hearing restoration.

## Data Availability

Data available on request to the corresponding author.

## Abbreviations

PSA	persistent stapedial artery
TORP	total ossicular replacement prosthesis
CT	computed tomography
MRA	magnetic resonance angiography
MRI	magnetic resonance imaging
PG	perichondrium graft
TM	tympanomeatal flap
I	incus
SA	stapedial artery
FN	facial nerve
C	cochleostomy
P	promontory
ABG	air-bone gap
